# Supplementary material for: Brain mechanism of unfamiliar and familiar voice processing: an activation likelihood estimation meta-analysis
Source: PeerJ. 2023 Mar 13;11:e14976. doi: 10.7717/peerj.14976 (PMC10019337; doi:10.7717/peerj.14976)
Supplement: Supplemental Information 2 [file peerj-11-14976-s002.docx]

First, it deals with an important topic, namely the brain mechanisms for unfamiliar and familiar voice identity processing. The neural basis of voice has received a large amount of exploration and confirmation since Belin et al. (2000) first discovered the voice-selective sensitive region in the human brain. Meanwhile, the importance of voice identity for interpersonal communication is self-evident, as we judge the familiarity of the speaker based on the voice identity information, and then determine the communication mode. Yet despite the wealth of research on voice, the study of voice identity is relatively ‘young’. To be specific, the terms used in voice identity processing with different levels of familiarity is unclear and confusing, as well as the experimental task design is difference. This research status leads to the current brain neural mechanism of speaker identity processing based on voice cannot effectively distinguish differences in voice familiarity.

Second, our approach is comprehensive and our methods are reliable. The present study based on the different clearly defined levels of voice perception, "voice identity perception" and "voice identity recognition/identification", and then adopted the meta-analysis method of activation likelihood estimation (ALE) to summarize the similarities and differences in brain mechanisms between unfamiliar voice identity and familiar voice identity processing. We believe that this approach can effectively integrate existing studies and provide a neutral assessment.

Third, to the best of our knowledge, the current study could provide a clear definition of these concepts and a reference for brain mechanism patterns for subsequent research on voice identity processing.
